# Transoral Laser Microsurgery in Early Glottic Lesions

**DOI:** 10.1007/s40136-017-0148-2

**Published:** 2017-03-11

**Authors:** EV Sjögren

**Affiliations:** Department of ENT and Head and Neck Surgery, Leiden University Medical Center, Albinusdreef 2, 2300 RC Leiden, The Netherlands

**Keywords:** Early glottic carcinoma, Early glottic cancer, Tis–T1–T2, TLM, Laser, Voice outcome

## Abstract

**Purpose of Review:**

To give an overview of the evolvement of transoral laser microsurgery (TLM) in the treatment of early glottic carcinoma and highlight the contribution of recent literature.

**Recent Findings:**

The indications and limits of TLM have been well specified. Effects on swallowing have been well documented. Introduction of narrow-band imaging (NBI) and diffusion-weighted magnetic resonance has been shown of additional value for outcome. The first reports on transoral robotic surgery show that it may be of added value in the future.

**Summary:**

TLM for early glottic carcinoma (Tis–T2) has very good oncological outcomes with indications of higher larynx preservation in TLM than that in radiotherapy. The anterior commissure is a risk factor if involved in the cranio-caudal plane, and reduced vocal fold mobility is a risk factor when this is due to arytenoid involvement. The best voice results are achieved when the anterior commissure can be left intact along with part of the vocal fold muscle although even in larger resections, patient self-reported voice handicap is still limited.

## Introduction

Early glottic squamous cell carcinoma (Tis–T2) is a highly treatable disease by any of the main treatment modalities available. With total laryngectomy as ultimate salvage, it carries a good prognosis for disease-specific survival regardless of the initial treatment strategy [1–5]. An important goal of initial treatment, other than survival, is therefore functional organ preservation by optimizing local control while avoiding total laryngectomy. Secondary parameters such as functional outcome (voice, swallowing, and airway), morbidity, and quality of life also play a large role when choosing treatment strategies, and in today’s healthcare systems, costs of treatment must also be incorporated.

The two main treatment modalities for early glottic carcinomas (Tis–T2) are organ sparing surgery in the form of transoral laser microsurgery (TLM) or open partial laryngectomy (OPL) and radiotherapy (RT). They are all used both in primary treatment and in the treatment of recurrent disease. There is no formal proof that one treatment is more effective than the other including a recent Cochrane analysis [2•, 4, 5] although there is evidence that TLM as a primary treatment modality is associated with a higher laryngeal preservation rate than RT in early glottic carcinoma [6–9], and though it was not randomized, the study by Mahler was well controlled [6]. Therefore, treatment strategy depends on surgeon and patient preference and varies between countries, institutions, and individual surgeons. Advantages of TLM over alternative modalities include shorter hospitalization, lower morbidity, and superior functional outcomes compared to those of OPL [10••, 11••] and shorter treatment time and less damage to healthy tissue compared to those of RT. Also, an important advantage of using TLM as a primary treatment strategy is that it leaves open all treatment options in patients with recurrent disease. This may explain the fact that the studies mentioned earlier have found lower laryngectomy rates in cohorts of patients initially treated with TLM. TLM has also been shown to be more cost-effective than OPL or RT [12–16]. Because of the above-mentioned advantages, TLM has gained widespread approval and has now replaced OPL as the primary surgical modality. And although RT has traditionally been the gold standard of treatment for Tis–T2 glottic carcinoma, many clinics, especially in Northern Europe and the USA, have now adopted TLM as the primary treatment modality for early glottic carcinoma. This review focuses on the outcome of TLM in current treatment of precancerous lesions and early glottic carcinoma (Tis–T2).

## TLM: History, Concepts/Technique, and Oncological Outcome

### History of TLM

TLM as a procedure was first introduced in 1972 by Strong and Jako when they coupled a carbon dioxide laser to a surgical microscope [17]. Although initially used by many to treat small glottic lesions with reports showing high rates of local control (90–94%) and low rates of laryngectomy (0–4%) in selected midcord T1a carcinoma [14, 18–20], the technique was also developed further in the 1980s and 1990s by Eckel and Steiner among others. In his 1992 publication, Eckel reports on 110 patients treated with TLM from 1986 onward of which 80 were glottic tumors (Tis 8, T1a 28, T1b 8, T2 31, and T3 5). He also presented a classification system of resections (type I–IV), which forms the basis of the classification system used by the European Laryngological Society (ELS) today [21]. In the following year, Steiner published a series of 159 patients treated with TLM between 1979 and 1985 (29 Tis, 96 T1, and 34 small T2 tumors with mobile vocal fold) in which he reported a recurrence rate of 6% for this group and a disease-specific survival of 100% [22]. These two reports were the first to show that TLM could be used to safely remove larger lesions extending beyond the membranous true vocal fold to other laryngeal structures such as the arytenoid, anterior commissure, and the sub/supraglottis (T2–T3) laying the basis for current concept of TLM in laryngeal cancer.

### Concept of TLM, Oncological Outcomes, and Limitations

Contrary to open surgical procedures where the dissection involves removing anatomical structures following standard surgical routes, TLM is based on following the actual tumor spread itself, resecting only the involved tissue and structures using narrow margins to spare uninvolved structures and therefore improve functional outcomes. In doing so, TLM as a technique aims to remove the tumor by applying a phonosurgical narrow cancer-free margins resection for early glottic lesions (with optimization of functional vocal outcomes) or through a more aggressive compartmental resection potentially involving larger portions of the larynx in case of intermediate or advanced disease [23, 24••]. The ELS classification system for TLM resections is most widely used today taking into account both the depth and surface area of the resection [25, 26]. Small, midcord tumors can be resected en bloc. More extensive vocal fold lesions are usually removed piecemeal where transection of the tumor allows for a more adequate assessment of depth of involvement and for better exposition and more access during removal of the tumor. It is often advisable to resect (part of) the false vocal fold and/or petiole of the epiglottis to improve exposure. The overwhelming majority of surgeons use the CO_2_ laser for TLM. Its wavelength (10,600 nm) is absorbed by water, which allows for both cutting and ablation modes. Vessels smaller than 0.5 mm are coagulated which regulate bleeding. With the improvement in technical aspects such a scanner applications and pulsed delivery of the laser beam, adverse tissue effects have been reduced and precision increased in the past decades.

Following this concept, several large studies of consecutive patients published between 2000 and 2010 have shown that TLM provides excellent oncological outcomes with very high disease-specific survival for all stages of early glottic carcinoma with local control and larynx preservation being the highest in Tis and T1 tumors [27–31]. The outcomes of these studies are summarized in Table 1 to give an impression of the outcome that is achieved in large series with limited selection bias where TLM is performed by experienced surgeons. In the case of updated series from the same group, only the last series has been included.

**Table 1:** Summary of outcomes in large series of consecutive patients [30–32]

T category	Local control (%)	Ultimate local control with laser alone (%)	Larynx preservation (%)	Disease-specific survival (%)
Tis	70–94	94	99–100	100
T1a midcord	90–94	–	96–100	99–100
T1	85–87	93–95	94–98	99
T2	66–82	79–86	82–95	98

### Outcomes of TLM in Recent Years

A number of additional larger reports (>100 patients) on oncologic outcomes in early glottic carcinoma have been published since 2010 [33–38]. Most of them are in line with the findings of the larger series mentioned earlier. In some studies, outcomes are outside the ranges shown above, in these cases mostly by a few percentage points. Larger variances are mainly seen in studies with <20 patients within a T categories, in which one single aberrant case will shift outcome significantly [39–41]. Aside from random variations due to small numbers, positive deviances may also indicate selection bias with the most favorable lesions being chosen for TLM whereas the larger studies included in the table above tend to consist of (near) consecutive patients. Therefore, it is hard to determine the value of deviant findings. Incorporating all these studies in a table such as the one above would lead to large ranges in outcome making interpretation difficult or even meaningless. Therefore, as stated above, the outcomes in Table 1 should be viewed as what is attainable in large series of (near) consecutive patients treated by experienced surgeons.

### Limitations of TLM

Current limitations for TLM resection of glottic lesions are well summarized and discussed by Peretti in a recent paper from 2016 [24••]. He points out that reasonably high rates of success in terms of oncological and functional outcomes can be achieved for superficial lesions of the entire larynx with limited deep invasion towards the anterior and lateral visceral compartments. Involvement of the posterior paraglottic space with encroachment of the cricoarytenoid joint and infiltration of the laryngeal framework negatively influence both oncological and functional outcomes, and the role of TLM here is limited to anecdotal cases. In the case of reduced vocal fold, its mobility is therefore important to ascertain whether this is due (only) to muscle infiltration and tumor bulk or if there is actual fixation of the joint as these two entities carry a different prognosis. For patients with lesions of the anterior commissure extending to the supra- or sublgottis in the cranio-caudal direction, there is a higher rate of local failure but in this case, salvage therapy in the form of open surgery or non-surgical treatment is not hampered by previous TLM. In another recent and insightful review of TLM from 2015, Hartl points out that a definite prerequisite for TLM is maintaining the cricoid cartilage as an unstable airway structure leads to laryngeal stenosis and permanent tracheotomy. Because of impossibility of obtaining sufficient margins while maintaining a patent airway postoperatively, the need for cricoid cartilage resection is a contra-indication for TLM. Also, at least one cricoarytenoid unit (cricoid, arytenoid, cricoarytenoid joint/muscles, and associated recurrent nerve) must be spared to preserve laryngeal sphincter function. According to the author, it is generally accepted that prophylactic treatment of the radiologically classified N0 neck is not necessary as the rate of occult neck metastasis for early glottic cancer is less than 10% [42].

## Factors Affecting Oncological Outcomes of TLM

Although TLM is recognized as a standard treatment for Tis–T2 glottic carcinoma, controversy still exists as to the predictive factors for recurrence which may warrant revision after TLM or treatment by an alternative or additional modality [43]. Factors that have been identified as prognostic for a poorer outcome are T category, vocal fold fixation, arytenoid involvement, lateral extension into the bottom and roof of the ventricle with or without false cord involvement, anterior commissure (AC) involvement, and positive resection margins [40]. However, as Hoffman points out in a well-detailed discussion on the subject, currently, clinical risk stratifications for many of these factors remain inconsistent [43]. Some of the most common risk factors are discussed below.

### Definition of Early Glottic Carcinoma, and Tumor Category and Subcategory

There is no clear clinical definition for early glottic carcinoma. Some authors take it to include Tis and T1 lesions only, others define it as also including limited T2 lesions with a mobile vocal fold and an N0 neck, and others yet include all T2 lesions. Therefore, care has to be taken as to the specific make-up of the population of a study when evaluating the results.

Even with a fixed definition, there are still issues. As the TNM classification for T1 tumors only considers superficial spread and does not take into account the depth of a lesion, patient populations can still be heterogeneous depending on the individual interpretation of the TNM staging by the surgeon. Furthermore, as Ferlito points out along with DeSanto and Olsen, not all T1 lesions will behave in a similar fashion. Large T1 lesions may as a group behave more aggressively and follow a course more reminiscent of a higher T2/T3 category [44, 45]. This is illustrated by our own findings showing that when favorable T1 lesions (superficial midcord) are selectively treated with TLM, the outcome after RT for the remaining larger T1 lesions is less favorable than would be expected for an entire group of T1 glottic carcinoma treated with RT [46]. It is important to realize that also with the alternative treatment of RT, there is a subgroup of T1 glottic carcinomas that show a poorer response reminiscent of T2 lesions. This may be due to differences in biological behavior but the lack of clear staging criteria for depth infiltration and the resulting variation in staging between T1 and T2 tumors may also contribute to this phenomenon. Early lesions on the free edge of the vocal fold have a tendency to remain confined to the lamina propria, superficially involving the AC and/or the vocal process of the arytenoid. Deep extension is limited by the vocal ligament, which reduces the possibility of vocal muscle infiltration [29]. In the author’s opinion, these lesions are true T1 lesions rendering the favorable prognosis expected of a T1 tumor. Any gross involvement of the vocal fold muscle—regardless of normal mobility—should therefore be considered a T2 tumor necessitating larger resection margins and more rigorous follow-up including imaging as the chance of a submucosal recurrence is higher than that in superficial lesions. Care should be taken to assess depth of infiltration both at the free edge and in the floor of the Morgagni sinus. As there is no ligament present here, anything but superficial involvement of the mucosa will involve the thyroarytenoid muscle and should therefore be considered a T2 tumor. Interestingly, in 2011, Lucioni published his results both according to TNM classification and to ELS resection type, which is more reflective of the true extent of the lesions. Such a classification may ultimately be of additional value in assessing prognosis in TLM of early glottic carcinoma [41]. Despite of the above confounders, current TNM classification (Tis, T1, and T2) has been found to significantly affect prognosis in TLM of early glottic carcinoma [28].

The same is true for the T1 subcategories T1a (localization on one vocal fold) versus T1b (localization on both vocal folds) as well as the discarded subclassification for T2 tumors in T2a (normal mobility of vocal fold) and T2b (impaired mobility of vocal fold) as a number of recent studies have highlighted. Lucioni found a local control with primary laser of 90% for T1a (*n* = 124) and 81% for T1b (*n* = 37). Ultimate local control with laser alone was 98 and 90%, respectively, and larynx preservation rate was 98% in both cases [41]. In 2015, Canis published the 27-year experience (1979–2006) with an impressive 404 cases of T1a glottic carcinoma of the Gottingen group. Local control was 86% and larynx preservation was 97% with a disease-specific survival of 98% [47•]. A review of 16 studies on local control after TLM by O’Hara published in 2013 confirms this rate of local control for T1a (88%, *n* = 985) but finds that T1b tumors have a lower local control rate, although the study population is also smaller (78%, *n* = 187) [48]. As for T2 tumors, Canis again published a very large cohort in 2014 detailing their experience with T2 glottic tumors treated between 1979 and 2006 divided into 142 T2a (mobile cord) and 127 T2b (fixed cord). The 5-year local control was 76% for T2a tumors and 57% for T2b tumors. Larynx preservation was 93% in T2a patients and 83% in T2b patients with a disease-specific survival of 93 and 84%, respectively [49•]. In conclusion, these numbers show that there is a trend for lower local control within the T1 category for T1b versus T1a as well as for lower local control, larynx preservation, and disease-specific survival within the T2 category in T2b patients compared to that in T2a. Although officially the T2a and T2b subcategories are no more part of the UICC or AJCC staging systems, it is important to realize that impaired mobility of the vocal fold carries a poorer prognosis in TLM. As stated earlier, this poorer prognosis seems to be associated mainly with tumor infiltration of the posterior paraglottic space resulting in fixation of the arytenoid joint and not with impaired mobility from vocal fold muscle infiltration or pure tumor bulk. Peretti did not find an impact on local control for the latter, and in making this important distinction, he argues that in these cases, a cordectomy extended to the entire paraglottic compartment has been shown to be an oncollogically safe procedure [29].

### Anterior Commissure Involvement

Anterior commissure (AC) involvement has often been argued to be a risk factor for recurrence because of the particular anatomy of this region. In the past, some studies have found it to be a risk factor for recurrent disease [50–53], and others have not [29, 54–56]. Several recent studies have investigated the anterior commissure as an independent risk factor for local recurrence [43, 57–60]. Notably, in a large study from 2009 published by Rödel (463 T1–T2), analysis showed involvement of the AC to be associated with decreased local control in T1a and T1b but not in T2 tumors although survival was not affected. The author argues that TLM is still an effective treatment for tumors located in the AC as most of the recurrent tumors could still be treated with further TLM procedures [57]. Conversely, Hakeem found local control significantly lower in T2 patients with AC involvement but not in T1a or T1b, although there was a trend for poorer local control in T1b tumors in his study of 296 T1–T2 patients [60]. Laryngeal preservation rate and overall survival were not affected. Hoffman found patients with AC involvement to have lower local control, laryngeal preservation rate, and disease-specific survival in her study of 201 patients with Tis–T2 glottic carcinoma [43].

The discrepancies found between studies may be due in part to discrepancies in staging. Peretti highlights [24••] that it is important to differentiate between tumors located in the AC in the horizontal plane and tumors extending from the AC in the vertical plane to the supra- and/or subglottis. At the level of the true vocal folds, the thyroid cartilage is situated directly underneath the vocal folds which attach to the cartilage by Broyles’ ligament formed by the union of the thyroepiglottic ligament and the attachment of the vocal ligaments and consisting of dense fibroelastic tissue without glandular structures, blood, or lymphatic vessels [29]. Some authors argue this to be a weak point where tumor easily penetrates the cartilage transforming a T1 tumor into a T4 [43, 61]. Conversely, others have emphasized that Broyles’ ligament protects the cartilage and that therefore, tumors with superficial extension in the AC, especially in the horizontal plane (T1), rarely show thyroid cartilage infiltration [29, 62, 63]. This is in line with the author’s own experience where a past inventory at our institution of T1 tumors with anterior commissure involvement did not reveal any cases of hidden cartilage invasion (unpublished data). Peretti argues that tumors with pure horizontal spread in the AC form a good indication for TLM. The risk of poorer local control arises from tumors with vertical extension from the AC as these tumors have a close relationship with the underlying visceral spaces (pre-epiglottic space and subglottis) and carry a risk of microscopic spread into these areas. In support of this reasoning in which he highlights this important distinction, he found a negative impact on local control only for those tumors crossing the anterior commissure in the vertical plane [29]. Most authors agree that there is no absolute contra-indication for TLM for transcommissural tumors in the vertical plane although they emphasize that proper assessment of tumor extent using 70-degree-angled endoscopes, thin-slice (1 mm) CT, or MR imaging in anything but superficial lesions as well as complete exposure of the operating field, wide resection margins and advanced surgical skills are mandatory when dealing with tumors in this anatomic location to avoid understaging and possibly undertreatment [24••, 29, 58]. In a detailed report on AC involvement in T2 lesions, Blanch emphasizes the importance of a wide excision in a horseshoe shape with a top-down approach which systematically includes the resection of the base of the epiglottis, the ventricular folds, and some of the inferior pre-epiglottic fat when dealing with these tumors in their extensive report on AC involvement in T2 lesions. This superior wide approach enables a better identification of depth of infiltration and facilitates subsequent fiberendoscopic control. Notably, the author also found recurrence after TLM of the anterior commissure to be significantly related to the learning curve of the surgeon [58]. The ELS type VI resection was introduced in 2007 in a revision of the original classification (type I–V) specifically to deal with resections of the AC [26].

### Resection Margins

In TLM of early glottic carcinoma, it is generally accepted that a proportion of patients with positive margins will not develop a recurrence, and the relationship between resection margin status and relapse rate is still unclear. Difficulty in assessment of pathological specimens is a particular problem in TLM because of small sample size further compromised by carbonization, retraction, and issues with orientation. Also, studies on the role of resection margins in predicting recurrence after TLM in early glottic carcinoma vary in their recommendations. At the glottic level, most authors consider 1–2 mm as an adequate margin although some authors have suggested that margins less than 0.5 mm may be enough in Tis–T1 carcinoma as the ligament still forms a barrier to tumor spread in early lesions (Table 2). Although we accept very narrow margins in Tis–T1 lesions, the author believes that care should be taken to realize margins at the higher end of the spectrum once the tumor has spread beyond the ligament. In accordance with this, Canis reported obtaining margins of 2–3 mm in T2 tumors [49•]. Authors also differ in their handling of positive or inadequate margins. As shown in Table 3, four authors chose a complete wait and see policy [64–67]. Others preferred a wait and see attitude but performed several second-look procedures, on clinical indication [68–70] or using the site of the positive margins (deep versus superficial) to decide whether a patient should be re-excised or not [32]. Re-treatment with either TLM or radiotherapy was standard only for three authors [71–73], although not all patients were handled according to protocol, mainly because of patients’ preference. As a result, there were two studies in which positive/inadequate margins were handled with re-treatment in the majority of cases [69, 72].

**Table 2:** Studies on margin status in TLM for early glottic carcinomas

Tumor stage	First author (year published)	Number	Free margins defined as (mm)	Positive or inadequate margins (%)	Action on positive or inadequate margins
Tis–T1	Hartl (2007) [68]	79	>2	32	9%: re-excision 91%: FU
	Sigston (2006) [64]	52	>0.5	35	100%: FU
Tis–T2	Ansarin (2009) [72]	274	>1	34	38%: re-excision 30%: RT 32%: FU
	Grant (2009) [69]	49	NA	31	60%: re-excision 13%: RT 27%: FU
	Mortuaire (2006) [65]	110	NA	24	100%: FU
Tis–T3	Peretti (2010) [28]	595	>1	50	24%: re-excision 76%: FU
T1	Michel (2011) [70]	64	>1–2	38	4%: re-excision 96%: FU
	Manola (2008) [73]	31	>1–2	29	22%: re-excision 11%: RT 66%: FU
	Brondbo (2007) [66]	171	>1–2	36	100%: FU
T1–T2	Fang (2013) [67]	75	>1–2	51	100%: FU
	Crespo (2006) [71]	40	NA	20	25%: RT 75%: FU

**Table 3:** Table 3 Fraction of cases in literature receiving re-treatment and having a positive or inadequate surgical margins after TLM for early glottic carcinomas and the association with recurrence rates and mean recurrence rates

Re-treatment (%)	First author	Recurrence (%)	
0	Sigston Mortuaire Brondbo Fang	12 20 10 12	13.5
68–73	Ansarin Grant	10 8	9

Table 3 represents the fraction of cases receiving re-treatment in association with the total fraction of recurrences.

The average recurrence rate reported by authors adopting a wait and see attitude appears to result in a slightly higher chance of recurrence (mean of 13.5%), as opposed to that of those who choose the more aggressive approach of re-treating most patients (mean of 9%). Despite this, it has been argued that instead of solely assessing the surgical specimen, an experienced intra-operative opinion on how radical the surgery was is deemed an important factor in decision-making. Sigston advocated a “wait and see” policy, pointing out that because of the readily accessible localization of glottic cancers, qualitative visual follow-up is adequate for monitoring patients. Moreover, the glottic area generally reveals symptoms earlier than other areas, enabling earlier detection, as patients will visit their physicians outside of protocol if they experience symptoms [64]. Sigston and coworkers found that 84% of their patients with positive surgical margins would potentially have received unnecessary additional treatment without this wait and see policy [64]. Later authors have also adopted this point of view [65, 66, 68, 69]. At the author’s institution, we agree with this reasoning although we adopt a more aggressive policy in positive deep margins, especially in larger T1 and T2 tumors as the chance of a submucosal recurrence is larger in these cases. As for second-look microlaryngoscopy, the recent ELS recommendations on follow-up after treatment for laryngeal cancer take the position that a second-look microlaryngoscopy is mandatory in case of positive margins and can be indicated in various other situations such as close margins, granulomas, web formation, or involvement of certain laryngeal subsites such as the AC, the laryngeal ventricle, and the subglottis [74].

## TLM in Salvage

TLM is used in the treatment of recurrent disease either after initial TLM or after RT. However, the local control of salvage TLM is lower than that in primary surgery. Peretti found that the pathway of spread in the recurrent carcinoma is the most important factor in predicting its endoscopic curability. He classified 58 patients according to the location of the recurrence compared to the initial site of the tumor. Salvage TLM was possible in 86% of patients where the recurrence occurred in the same area, and in 74% if the recurrence occurred in adjacent sites with superficial spreading or multifocal distribution. In the case of submucosal recurrence with spread in the visceral spaces of the larynx or cartilaginous framework, no patient was cured endoscopically [29]. Recently, Rödel reported a 5-year local control and larynx preservation rates of 64 and 91%, respectively, with repeated TLM in patients with early recurrence (rTis–rT2N0) initially treated for early glottic carcinoma (pTis–T2). For advanced recurrence (rT3/T4aN0/N+), these were 28 and 75%, respectively. The authors conclude that in case of early local recurrence after primary TLM for early glottic carcinoma, further TLM seems to be justified as an organ-preserving treatment option. In contrast, salvage laryngectomy should be considered early for patients with advanced local or loco-regional recurrence [75]. Huang analyzed 50 rT1–T2 lesions treated with salvage TLM and found local control to be 70% with a larynx preservation of 88% and disease-specific survival of 98%. A second recurrence after salvage TLM significantly impacted larynx preservation and disease-specific survival causing the author to argue for oncological safe resection margins in salvage TLM rather than sparing healthy tissue for a good voice outcome [76].

## Recent Advances in TLM-Related Areas

### Optical and Molecular Imaging Techniques

To improve tumor detection and tumor margin identification, a number of optical and molecular examining technologies have been developed over the last few years. These technologies can be divided in those that improve superficial detection of tumor extent in the superficial plane such as fluorescent imaging, contact endoscopy, and narrow-band imaging and those that aim at detecting extension in the deep plane such as optical coherence tomography, confocal endomicroscopy, and Raman spectroscopy. One of the most implemented techniques in clinical practice so far is narrow-band imaging (NBI). This is an endoscopic technique that uses selected wavelengths of light to enhance the vascular pattern of the mucosa. Superficial carcinomas are better identified due to their aberrant vascular pattern. This technique has been shown to increase diagnostic accuracy in terms of better definition of surgical margins, tumor staging, and early detection of residual or recurrent disease as compared to ordinary white light endoscopy [77]. Piazza found that 18% of patients had a diagnostic gain by using NBI compared to routine white light endoscopy [78]. Bertino found a diagnostic gain of 11% in terms of margin definition and 18% in terms of suspicion of malignancy [79]. Kraft found accuracy to increase from 90 to 97% when using NBI as opposed to routine white light endoscopy [80]. Finally, Garofolo found that when using NBI to guide resection margins, the rate of positive superficial margins was 3.6% as opposed to 23.7% in a control group [81]. The diagnostic advantage afforded by NBI has also been shown to apply after radiation and/or chemotherapy [82, 83]. In their recommendations for the follow-up of laryngeal cancer, the ELS recently stated that incorporating NBI into postoperative surveillance will give more accurate follow-up [74]. Despite these advances in diagnosing superficial extension of early glottic lesions, the challenge of depth resolution still remains, and there is as of yet no routine clinical application to guide the depth of TLM resection in the upper layers of the vocal fold in superficial lesions. Technologies such as optical coherence tomography, confocal endomicroscopy, and Raman spectroscopy are being developed but have not yet been introduced in daily clinical practice. Routine imaging (CT and MR) is invaluable in assessing tumor involvement of deep structures such as the paraglottic and pre-epiglottic spaces, laryngeal framework, and extra laryngeal soft tissues but are as of yet not detailed enough to guide narrow-margin surgery of the lamina propria and superficial vocal fold muscle.

### Conventional Imaging Techniques (CT/MR)

Accurate clinical staging of early glottis carcinoma is critical to therapeutic planning and for choosing between organ preservation strategies. Involvement of the visceral spaces of the larynx (paraglottic and pre-epiglottic spaces) and laryngeal cartilage framework must be ascertained to differentiate early glottic tumors—particularly T2 lesions from more advanced categories (T3–T4). Also, during follow-up, although superficial recurrences can be detected by endoscopy, submucosal recurrences which are frequently observed after TLM [24••] are more difficult to detect and may spread within the deep visceral spaces of the larynx before they are bulky enough to be detected by endoscopy. So in both diagnosis and follow-up, imaging plays a crucial role in all but superficial T1 lesions. In the work-up of laryngeal cancer, CT is currently the mainstay in the assessment of submucosal tumor spread. However, MR has recently been shown to be more accurate in staging of T1–T2 glottic carcinoma (MR 80% accurate versus CT 70%) [84]. Moreover, new MR techniques such as diffusion-weighted MR have also been shown to offer a more precise definition of signals differentiating tumor, edema, and scar. When clinical strategy depends on the precise local mapping of primary or relapsing tumor (such as in ascertaining the indication for TLM), MR can provide information that is presently beyond the capability of CT [85•] although evaluation of MR needs a higher level of experience and interdisciplinary collaboration [84].

### Alternative Laser Types

In recent years, alternative lasers have been introduced to treat early glottic carcinoma. Using different wavelengths affecting different chromophores of the tissue than the standard CO_2_ laser, their main advantage is increased preservation of the vocal fold soft tissue including mucosal superior lamina propria (SLP) in more superficial tumors leading to superior functional (voice) outcomes and shorter wound healing times. Also, most of them do not require straight beam delivery and are used as flexible fibers. In 2013, Murono obtained local control in 22 out of 24 patients with T1 glottic carcinoma treated with potassium-titanyl-phosphate laser (KTP). In 2014, Zeitels published his series of 117 patients (T1–T2) treated with KTP laser. Local control for T1 and T2 lesions was 96 and 80%, respectively. Fifty percent of recurrences were controlled with RT while the other 50% failed RT and died of their disease. Disease-specific survival was 99% for T1 and 89% for T2 [86]. This shows that at least in T1 carcinoma, KTP may be a valid alternative to traditional CO_2_ laser. The disease-specific survival in T2 is lower than that expected with CO_2_ laser. In 2014, Cömert showed that diode laser was as effective in treating 72 patients with T1–T2 glottic carcinoma as RT (local control diode laser 93% versus RT 90%) [87].

### Transoral Robotic Surgery

Recently, transoral robotic surgery (TORS) has emerged as an innovative technique in the surgical treatment of head and neck cancer. For this technique, an angled vision endoscopic camera is introduced transorally through a mouth gag to provide a magnified view of the pharyngo-laryngeal structures. The surgery is performed with instruments that allow scaled and tremor-filtered movement. This technique results in wide tissue exposure that facilitates multiplanar dissection. Additionally, natural hand tremor is filtered out, large hand movements are adjusted, and the surgeon is given true depth perception by the 3D visualization of the surgical field [88•, 89]. Previous studies have demonstrated the value of this technique for other sites of the upper aerodigestive tracts but the role of TORS among the different treatment options for early glottic cancer is still not clear as the technique was not initially developed for this application [88•]. Recently, it has been shown that combining TORS with CO_2_ laser fiber is feasible allowing for precise cutting with a flexible tip and effective hemostasis of vessels 0.5 mm or less [89]. Also, a preliminary study of the feasibility of TORS in T1–T2 carcinoma of the glottic and supraglottic larynx has been published [88•]. This study which is the first report on TORS in T1–T2 glottic carcinoma showed that although TORS is made difficult by the same limitations as TLM (short neck, prominent incisors, large tongue base, reduced mouth opening, and retrognathism), it is not necessary to get a direct vision of the laryngeal tumor in order to carry out surgery. In this study, it was possible to access two carcinomas of the anterior commissure that were not approachable by TLM. This may prove to be an added value of TORS to the current treatment options in early glottic carcinoma. Currently, the expectation is that in the coming years, TORS will be adapted to better suit endolaryngeal surgery with better exposure and finer instruments (Remacle, personal communication).

## Functional Outcomes of TLM

When looking at functional outcomes, the three main functions of the larynx potentially affected by any treatment are airway, swallowing, and voice. Of these, vocal function is the most frequently affected. Additionally, other acute or chronic complications induced by the operation as well as overall quality of life need to be considered.

### Voice

Many studies have demonstrated that the quality of voice after TML is related to the extent of the resection [90]. The general consensus in literature, as well as in the author’s own experience, is that type I–III resections, where at least some of the vocal muscle and the contralateral side of the anterior commissure are preserved, result in very acceptable voices with only mild dysphonia and low voice handicap reported by patients themselves [91–93]. Grades of dysphonia for these resections vary from less than 1 to 1.5 on the GRBAS scale (grade roughness breathiness asthenia strain) for perceptual rating corresponding to mild dysphonia [91, 94–96]. Voice handicap index (VHI) scores are very close to normal ranging from 19 to 28 where 120 is the maximal score and scores of 15 or under are considered normal [90, 92, 97]. Some reports on voice outcomes after type I–II resections do not even differ from normal controls [94, 95]. Conversely, Vilaseca found that on average, voice quality after TLM was worse than that in normal controls for every resection type but that in the case of type I resection, perceptual analysis showed it was normal in a majority (66%) of patients and as high as 55% even in type III resections [96].

However, wider excisions (type IV–VI) including total resection of the vocal fold muscle and/or the AC will lead to more severe forms of dysphonia in most patients. Perceptual grades of dysphonia reported in these groups vary from 1.6 to 2.7 [94–96, 98, 99] with most groups reporting scores of 2 or higher meaning moderate to severe dysphonia. The VHI in these patients is higher than that in more superficial resections but still relatively low ranging from 24 to 39 [90, 92, 98]. This relatively low score indicates that patients seem to accept their voice limitations. This is further supported by the fact that only 7% of 145 patients (T1–T4) were motivated for vocal fold reconstruction after TLM. Even in a subgroup of extended resections (type IV–V), this was only 16% [100]. Patients that do undergo reconstruction can expect improvement but not normalization of voice. Piazza reported a decrease in the VHI from 46 to 21 [101] and Remacle from 65 to 33 [100] in their reconstructed patients.

The data on vocal outcome from the past few years support earlier findings. Bahannan found that type I–III resections result in a significantly better voice outcome than type IV and V resections in 62 patients with Tis–T2 lesions. Fink reported a similar outcome in 49 patients with Tis–T1a lesions. Patients undergoing type I–III resections experienced on average an improvement in the VHI from 39 to 23 whereas patients undergoing a more extended resection had an increase in the VHI from 33 to 39 [92]. Mendelsohn found a postoperative VHI of 24 in 13 patients undergoing extended resections (type III–VI) with a mean grade of 2 (moderate dysphonia) [102]. The same author also published one of the only reports specifically on voice outcome after resections of the AC which showed a VHI of 37 and a mean grade of 2.1 (moderate dysphonia) in these patients [63]. Peretti reported on 89 type V resections (59 T2 and 30 T3 carcinomas). Voice outcome evaluation in a subsection of 48 of these patients 1 year after surgery revealed VHI to be 20, and perceptual dysphonia was mild in 82% and moderate in 18% of patients evaluated by the GRBAS scale [11••]. Additionally, two series looking at longitudinal outcomes highlight the fact that voice shows an initial decline after surgery but will improve [102, 103] and stabilize at 6 months after TLM [103].

Interestingly, in a publication from 2013, Hillel challenges the accepted correlation between resection depth and voice outcomes by raising the issue that patients undergoing type II resection may even have better voice outcome than those after type I resection, and that even thought the ligament could be spared, it should be resected in patients with only a minimal residue of SLP for functional reasons [104•]. A computer model published by Mau in 2015 provides theoretical support for this finding although the authors state that further clinical data are required to support this conclusion [105•].

### Swallowing

As for swallowing, there is a dearth of data for limited lesions. This may be because swallowing is an underestimated issue in these patients but it may also point to the fact that it is not considered an issue in clinical practice. In extended lesions, sporadic cases of severe long-term dysphagia are reported. In 2004, Bernal-Sprekelsen published a series of 45 T2 glottic carcinomas treated with TLM. The average duration of nasogastric feeding tube dependence was 0.18 days. Out of his 45 patients, only 1 underwent percutaneous endoscopic gastrostomy placement, tracheotomy, and finally, total laryngectomy for swallowing problems [106]. Two recent publications have contributed to detailing of swallowing after TLM. In a series of 89 type V resections, Peretti found favorable swallowing outcomes. In the MDADI (M.D. Anderson dysphagia inventory), self-evaluation questionnaire of swallowing patients scored an average of 96 out of a maximum of 100. Only 2 and 4% of patients showed tracheal aspiration on VEES (video endoscopic evaluation of swallowing) and VFS (videofluoroscopy) examinations [11••]. Age and T category were not found to impact these results in a second prospective study of swallowing outcomes in 40 patients with T2 carcinoma undergoing a type V resection, and Nasef found that only 12.5% of patients needed a nasogastric feeding tube which was removed after less than 5 days in most cases. The mean MDADI score after 3 months was 96, and none of the patients had incomplete laryngeal closure compared to 40% at day 1 after TLM. The mean duration of aspiration was 3.4 days [10••]. One important point to make is that pulmonary function in patients undergoing large resections, especially in recurrent tumors after RT where laryngeal functions may already be compromised, needs to be sufficient to withstand a period of aspiration before the sphincter function of the larynx is restored to avoid pneumonia.

### Airway and Other Complications

The upper airway and other complications are rare after TLM. The large series described under the oncological outcomes section reported the following complications: in 719 patients, Motta reported subcutaneous emphysema from penetration of the cricothyroid membrane in 5% of patients, which necessitated tracheotomy in four. An additional four patients required tracheotomy from oedema after cauterization bringing the total amount of tracheotomies to 1%. Bleeding resolved by endoscopic revision occurred in 1.6% of patients. In 595 patients, Peretti reported only major bleeding as a complication in 0.4% of patients in which endoscopic revision was sufficient to solve the problem. In 285 patients, Eckel reported one patient who developed a glottic stenosis and required further open surgery to restore an adequate airway. No other complications were seen.

### General Quality of Life

Quality of life (QoL) after TLM for early glottic carcinoma is generally good and does not differ significantly from normal controls. Recently, Vilaseca published two detailed studies on QoL in TLM. She found that 94% of 401 consecutive disease-free patients treated with TLM (including 254 T1–T2 glottic carcinomas) evaluated 1 year after treatment had a good QoL. RT and neck dissection had a negative impact on QoL whereas old age had a positive impact suggesting that older patients cope better with their disease. Relative voice impairment was detected especially in locally advanced tumor, and T classification (local early versus local advanced) and age were independent negative prognostic factors for QoL within the speech domain [107•]. In an earlier publication by the same author, 46% of patients considered speech as a relevant issue in QoL while only 31% scored under reference values. The author theorizes that the high expectations of minimal invasiveness created by TLM and the considerable percentage of patients that continue working without any official recognition of their disability may explain this discrepancy [108]. A third publication by Vals-Mateus shows that scores on voice and swallowing domains remain consistent over time and do not deteriorate [109•].

## Conclusion

TLM for early glottic carcinoma (Tis–T2) has very good oncological outcomes equal to those offered by other treatment options (OPL and RT) with some evidence that larynx preservation is higher in patients where TLM is employed as a primary treatment strategy as opposed to RT. Additional benefits of TLM are short treatment times, low morbidity and complication rates, and lower cost. T subcategories (T1a/b and T2a/b) have an impact on local control. Reduced mobility (T2b) only has an impact on local control if this is due to involvement of the arytenoid cartilage and not when this is caused by vocal fold muscle infiltration or tumor bulk. Involvement of the AC in the horizontal plane (T1) is not a risk factor for local recurrence whereas involvement in the vertical plane (T2) is. Voice outcome is near normal in limited lesions, and although dysphonia is moderate to severe in larger resections, patient-reported voice handicap is still limited. The best voice results are achieved when the AC can be left intact along with part of the vocal fold muscle. However, even after extensive resections, only a minority of patients will wish to undergo reconstructive phonosurgery, which can improve voice outcomes significantly. Swallowing is very rarely affected in the long-term even in extensive resections. TLM has a definite role as salvage treatment in early recurrent lesions both after initial TLM and RT, and with careful patient selection, ultimate disease survival is not compromised by salvage TLM. Introduction of NBI as an additional imaging modality into diagnosis, treatment, and follow-up has significantly improved disease detection and radicality of resections in the mucosal plane. There may be an additional role for TORS in the future in tumors that cannot be exposed by routine TLM and lasers such as KTP and diode as alternatives to traditional CO_2_ laser may have a role in sparing function if equal disease control can be maintained which seems to be the case in initial studies concerning T1 lesions.
